# Engineering for a clear image: a comparative focus on accommodation

**DOI:** 10.1038/s41433-024-03131-z

**Published:** 2024-07-13

**Authors:** David Williams

**Affiliations:** Department of Veterinary Medicine, University of Cambridge, Madingley Rd, Cambridge CB3 0ES and St John’s College, Cambridge, CB2 1TP England, UK

**Keywords:** Physiology, Visual system

## Abstract

The eye requires the ability to focus images near and far and throughout evolution numerous mechanisms have developed to allow this accommodation. From primitive organisms which use a small pupil to effect pinhole camera optics without a lens through more complex eyes with a lens that is moved antero-posteriorly along the visual axis or the shape of which is changed, the eye has engineered numerous accommodative mechanisms. Human inventors have developed cameras with remarkable accommodative abilities but none match the remarkable focussing abilities of the four-eyed fish Anableps or the cormorant which similarly manages to focus above and below water, to give just two examples from the animal kingdom, perfectly adapted to their environments and behaviours.

## Abstract (zh)

眼睛需要聚焦于远近物象的能力, 在进化过程中, 形成了许多机制来实现这种调节作用。从使用小瞳孔来实现无晶状体针孔相机光学器件的原始生物, 到更复杂的带有可沿视轴前后移动或改变形状的晶状体的眼睛, 眼睛已经产生了许多调节机制。虽然人类已发明出具有强大调节能力的相机, 但没有一种相机能够与四眼鱼Anableps或鸬鹚能够在水面上和水下同样聚焦的强大聚焦能力相媲美, 仅举动物界中两个例子, 这两种动物完全适应了其环境和行为。

## Introduction

If I asked you to design a camera that would be able to focus objects near and far you could, I guess, start with a pinhole camera. It has an infinite depth of field so keeps all images in perfect focus however near or far they are from the observer. The camera obscura uses just this technique and was described first, providing a pinhole image of the sun, in the Chinese Zhoubi Suanjing writings as far back as 1046 BCE. The problem is that this simple technique comes at a significant cost: it allows only a very low light input. Inserting a lens allows one to use a much larger aperture while still allowing images to be focussed. Such simple lenses were used in the earliest cameras produced by inventors such as Thomas Wedgewood [1] and Henry Fox Talbot [2] to name but two. But a single lens has a defined focal length. How do you aim to change this? Moving the lens backwards and forwards seems a sensible option, or manually changing lenses, though these days this seems rather a prehistoric option! Telephoto lenses, as developed first by Peter Barlow, used two lenses, a front positive imaging cell and a rear magnifying negative cell. A true zoom lens, able to focus near and far, was not perfected until the 1970s and involved a complex series of lenses together with the mechanisms to move them backwards and forwards [3]. More recently though, the tiny optics of miniaturised digital cameras have encouraged the development of lenses which can alter their focal distance by changing shape either with a droplet of liquid which alters on application of an electric charge [4], or a shape-memory alloy lens which changes shape again in response to an electric charge [5]. All these potential designs from the pinhole camera, through lens movement to shape-altering lenses are merely reflections of what animals have already achieved in nature to achieve optical accommodation as we shall see.

## Some basic concepts in comparative refraction

In all vertebrates, the image formed in vision is focused on the retina by two refractive components, the cornea and the lens. In terrestrial vertebrates, the cornea is the principal refracting surface as light passes from air to the aqueous humour in the eye and the difference in density and thus refractive index of air (refractive index 1) and aqueous humour (refractive index 1.34) gives rise to this substantial refraction [6]. The lens has a higher refractive index than that of aqueous humour but not to the degree of the difference between the two faces of the cornea. In aquatic organisms, however, the refractive power of the terrestrial cornea is lost and here the lens provides all the refraction the eye requires. The ratio of the focal length of the lens to the lens radius in such situations, Matthiesen’s ratio, is 2.55 and the lens has to be spherical to afford maximal refraction.

This is the also the case in small terrestrial mammals and small birds where given the diminutive size of their globes it is essential that the lens is spherical and takes up most of the volume of the eye. Having said that such small eyes do not need to be much concerned with accommodation, since the smaller the size of the lens, or strictly speaking the pupil aperture, the greater the depth of field and thus the lesser the need for accommodation. The equation for depth of field is 2u^2^Nc/f^2^ where u is the distance to the subject, confusingly N is the f number and f is the focal length of the lens while c is itself the circle of confusion! As the f number is the inverse of the aperture it can be seen that the smaller the pupil the greater the depth of field and the less the need for accommodation. Having said that good acuity needs a great illumination and so a wider pupil, hence the need for accommodation, whatever arrangement is used to achieve it.

## Primaeval accommodation

**Fig. 1:**
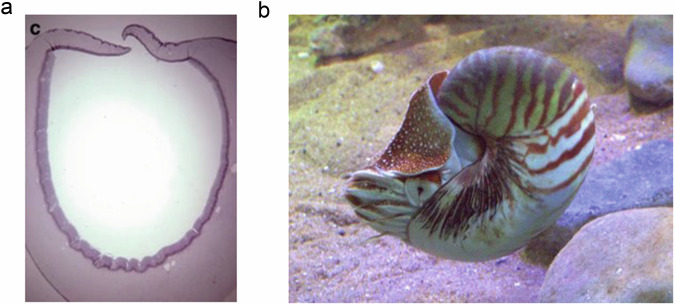
Histological image (left) of the eye of Nautilus (right) showing a small pupil allowing the eye to act as a pinhole camera avoiding the need for a lens to focus the image. Images courtesy of Professor R Dubielzig.

The earliest eyes, as I noted in a paper for the Cambridge Ophthalmic Symposium nearly a decade ago [7], were simple light-sensitive eyespots. These developed into photosensitive cups which could detect the direction of incident light, but to form an image a lens was needed. Or maybe not. Nautilus, a primitive cephalopod for which fossils exist as far back as the Triassic more than 200 million years ago, had then and has now neither cornea nor lens but a camera-style eye nevertheless [8] with a pinhole camera capable of producing an image (Fig. 1) [9]. In the fossil record we have no examples of the development of the lens, but molecular biological approaches show how alpha-, beta- and gamma-crystallins are key in the production of a transparent intraocular refractive structure, but one where the proteins also have anti-oxidant or chaperone functions. The key lens structural protein, alpha-crystallin, is a crucial chaperone molecular while other crystallins are small heat shock proteins. Different lens crystallins are expressed across the lens to change the refractive index of the lens across its diameter, thus reducing the problem of spherical aberration. James Clerk Maxwell first hypothesised that there should be such a gradient in fish eyes though it was Matthiessen who showed that this actually occurred in the piscine lens. This allows for spherical lenses with good optics but surely does not take us much closer to an understanding of accommodation and how eyes might be engineered to achieve it. Having said that these differences in refractive index across the lens might not only remedy spherical aberration where photons of different wavelengths refracts differently across different portions of the lens but also potentially gives a multifocal lens. We shall return to this intriguing possibility later.

## Piscine accommodation

As we have noted earlier, eyes under water generally have spherical lenses to maximise refraction given the reduced effect of the cornea as a refractive surface. This means that changing the shape of the lens is not possible. Some researchers have found that more primitive fish, the lampreys, accommodate by changing the curvature of the cornea with the extraocular *musculus cornealis* [10]. Others however suggest that the effect is actually to push the lens posteriorly [11]. The same researcher suggested that myopic accommodation in some lampreys was effected by contraction of extraocular muscles increasing the axial length of the globe. Having said that, retinoscopy of these animals underwater is taxing in the extreme. Handling of the fish itself may well change their refraction. Studies of free-swimming sharks demonstrate only the smallest excursions of accommodation. This is mediated by a papilla known as the pseudocampanule acting as a protractor lentis ‘muscle’, though its contractile element is provided by a thin sheath of contractile cells from the *pars ciliaris retinae* (Hueter 2001) [12]. Others have found a non-spherical multifocal lens which might change its focal length by lateral movement rather than motion along the visual axis [13]. More advanced teleost fish have a significantly better accommodative range provided by the *retractor lentis* muscle first discovered in 1834 in Wallace and by which the spherical lens is pulled caudally to focus more distant objects. In addition, many teleosts have the equivalent of a ciliary muscle but it is a tiny sliver of tissue and probably plays little part in accommodation in most species. In the tubular eyes of deep sea fish such as *Dissomma anale* and in the sandlance *Limnichthyes fasciatus* this is not the case. In these fish the muscle flattens the thickened cornea which provides at least 30% of the eye’s refractive power.

## Lower vertebrate accommodation

As far back as 1882 Hirschberg reported retinoscopic findings that frogs and toads are emmetropic in air while aquatic amphibian such as newts are emetropic in water. Their accommodative range is small maybe 2-4D and is achieved by contraction of a protractor lentis muscle on the ventral aspect of the lens. Anurans have a dorsal protractor lentis muscle as well as the ventral contractile fibres.

**Fig. 2:**
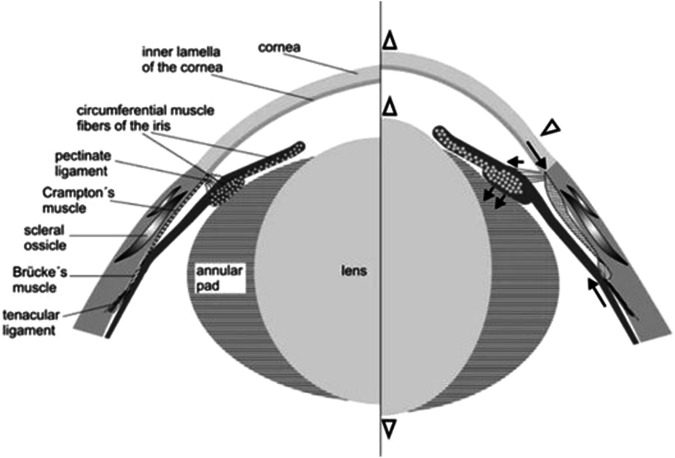
Diagram of accommodation mechanisms involved in the avian eye. From Ott [6].

Although we tend to lump lizards, snakes and crocodiles together as reptiles in reality they should be divided into two clades, first the Lepidosauria, lizards, snakes, amphisbaenians and the Tuatara and then their sister group the Archosauria including crocodiles, birds, extinct dinosaurs and probably also turtles. We might expect that their accommodative mechanisms would relate to their evolutionary position, but actually lizard and avian eyes are more similar while snake and chelonian methods of accommodation differ more because of their lifestyle than their evolutionary status. The lizard and bird eyes have ciliary muscles divided into anterior (Crampton’s) and posterior (Brucke’s) muscular portions. Crampton’s muscle reduces the radius of curvature of the cornea as it originates in the sclera beneath a supportive ring of scleral ossicles and inserts directly onto the inner lamella of the cornea. The changes in refractive power of the cornea, bulging outwards accounts for about 40% of the total accommodative power in chickens (Glasser 1984) while in hawks this can change the refractive power of the cornea between 2.8 and 6.5D (Glasset 1987). Bruck’e muscle inserts on the sclera but also extends backwards into the ciliary body, so pulling this forward and reducing the tension on the lens wulst. Deformation of the lens is produced by the circumferential striated muscles of the iris applying force to the lens wulst. This results in a mild anterior lenticonus. There are significant differences in this action between different avian species and similarly different lizard species vary in the degree to which corneal and lenticular changes influence accommodation. (Fig. 2).

Snakes lack a ciliary muscle altogether and have no scleral ossicles either. Changes in the shape of the lens in accommodation may occur but forward displacement of the spherical lens might be the primary mechanism of accommodation, this fitting with the theory that snakes were at some point aquatic organisms. Chelonia stands in both camps, as it were, with terrestrial and aquatic species as well as those inhabiting land and water. The European Pond turtle, for instance, active above and below the water level develops an anterior lenticonus to accommodate although it is difficult to know how ciliary muscles change the shape or the position of the les.

Given all we have said about accommodative mechanisms in other reptile groups, one might think that crocodiles, active above and below water might have similar accommodative mechanisms, but it appears that these species cannot accommodate to compensate for the difference in refraction above and below water, but they appear highly hypermetropic in water and emmetropic in air.

## Mammalian accommodation

We might think that avoiding the problems of accommodation under water, terrestrial animals would have eyes and accommodative powers similar to those of humans but as long ago as 1980 Sivak pointed out that it was inappropriate to assume that human accommodation mechanisms would be the same across all animal groups. It has to be said that many mammals do not need much in the way of accommodation. As I tell many owners of dogs and cats that I see with substantial visual compromise, as they neither read nor drive a car, acute vision is not as important to them as to we humans. Thus it is not surprising that dogs are estimated to have accommodative ability of no more that 2–3D [14]. While we do have data in the peer reviewed literature on the refractive status of domestic dogs and cats kept as companion animals [15–17], no detailed studies of accommodative ranges in these animals have been published barring the work of Hess and Heiner in 1898 [18]. These researchers suggested that the dog has a minimal range of only +/-1D and others found that the cat had a little more at 2–4D [19], although we must note that this was in a review paper without hard data. Instead, we have research papers on the accommodative ranges of the elephant [20] and rhinoceros (at around +/=3D) [21] and the Indian mongoose with an accommodative range as high as 11–13.5D [22]. Quite what this species does with such a high accommodative range is unclear, or how it changes its refraction so much.

It was thought for many years, following Walls’ seminal text of 1942 [23] that the horse has a passive accommodative mechanism resulting in lower field myopia. Several authors perpetuated the error that a ‘ramp retina’ mechanism existed by which the globe was aspherical with a shorter lens to retina distance for downgaze [24, 25]. Sivak and Allen disproved this anatomically in the post-mortem eye and by in vivo refraction 30 years later [26]. Harman and colleagues a further 20 years on demonstrated behaviourally that head movement allows the horse to maximise its limited binocular visual field rather than having a true myopic downgaze. All of which shows that retinoscopic research has to be seen in the context of the relevant animal’s behaviour. Prime examples of the link between behaviour and accommodation occur in the last examples of accommodation across the species as shown below.

## Extreme accommodation

What is the most extreme example of accommodation in the animal kingdom one might ask. Imagine the need to see a well-focused image above and below water. As we noted above, below the water level the cornea has no refractive power, while above it the cornea provides the main refractive element in the eye. A seal needs good visual acuity underwater to hunt its piscine prey but also must focus well when above water. The way in which is manages to attempt this refractive miracle returns to the mechanism that Nautilus adopts without a lens [27, 28]. The focussing power of its lens renders it emmetropic underwater while the pinhole camera effect of its miotic pupil above water, where the light intensity is much greater and so it does not need such a wide pupil aperture, means that the eye does not require refractive power to provide a focussed retinal image. This also means that the profound astigmatism of the pinniped eye, designed to optimise streamlining of the ocular surface, has no effect on vision above water, where it would be a significant impairment in a normal mammalian eye [29]. The refractive advantages of the extreme miosis of the pinniped pupil above water are significant disadvantages for the veterinary ophthalmic surgeon during cataract surgery in these species [30]. Intraoperative mydriasis is difficult to maintain and intracameral atropine and epinephrine are required throughout the phacofragmentation procedure [31].

**Fig. 3:**
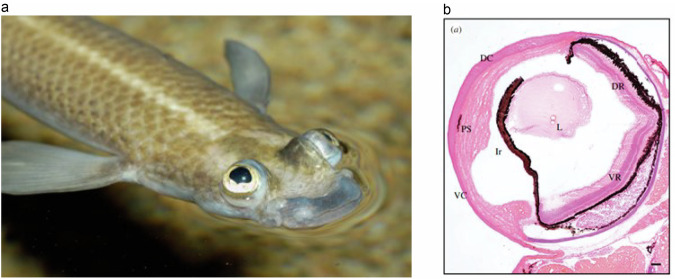
The eye of Anableps the ‘four-eyed fish’ (left) has a dorsal and ventral corneas and retinas with differing intraocular optics allowing the lens to focus light from above and below the water’s surface. From Perez et al. [32].

**Fig. 4:**
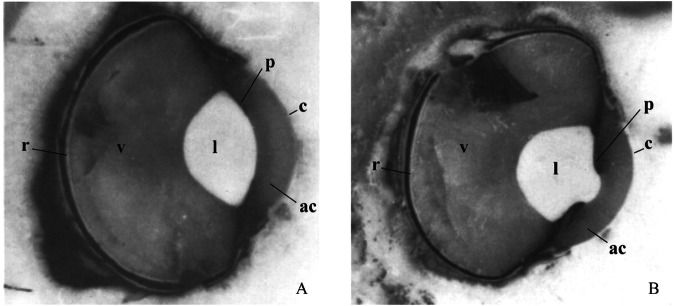
Extreme accommodation in the cormorant eye produced by iridal compression of the anterior lens. **a** (left) **b** (right) From Levy and Sivak [35].

A totally different solution to the problem of seeing above and below water comes with the four-eyed fish *Anableps* (Fig. 3) [32]. Here the optics of the portion of the globe seeing below water are totally different from the portion seeing above water rather like a photographer changes lenses in an old fashioned single lens reflex camera prior to telephoto lenses [33]. What then of a bird needing to see above and below the water level such as the cormorant? As early as 1909 Hess had documented that the anterior portion of the lens of the cormorant *Phalacrocprax carbo* is squeezed by the iris to bulge forward and produce an anterior lenticonus (Fig. 4) [34, 35]. This produces profound myopic lenticular refraction to cope with the loss of corneal refractive power as the bird enters the water. The sea otter has the same mechanism for seeing above and below the water level with an extreme lenticonus formed as the pupil protrudes through the pupil [28].

## Conclusion

The manner in which the eyes of different species allow accommodation are many and varied. This shows how the eye has engineered this variation in refraction from simple use of a small pupil to provide a pinhole camera, through mechanisms to move the lens within the eye, change the shape of the lens or indeed change the shape of the eye. We have ourselves engineered cameras to provide a variation in focus but in this we are only mimicking the amazing accommodation available in eyes of animals from the ancient Nautilus to the remarkable Anableps.
